# Lipid-dependent coupling of secretory cargo sorting and trafficking at the trans-Golgi network

**DOI:** 10.1002/1873-3468.13552

**Published:** 2019-07-30

**Authors:** Julia von Blume, Angelika Hausser

**Affiliations:** 1 Department of Cell Biology, Yale School of Medicine, New Haven, CT, USA; 2 Max Planck Institute of Biochemistry, Martinsried, Germany; 3 Institute of Cell Biology and Immunology, University of Stuttgart, Germany; 4 Stuttgart Research Center Systems Biology, University of Stuttgart, Germany

**Keywords:** diacylglycerol, secretory cargo sorting, sphingolipids, trafficking, trans-Golgi network

## Abstract

In eukaryotic cells, the trans-Golgi network (TGN) serves as a platform for secretory cargo sorting and trafficking. In recent years, it has become evident that a complex network of lipid–lipid and lipid–protein interactions contributes to these key functions. This review addresses the role of lipids at the TGN with a particular emphasis on sphingolipids and diacylglycerol. We further highlight how these lipids couple secretory cargo sorting and trafficking for spatiotemporal coordination of protein transport to the plasma membrane.

The trans-Golgi network (TGN) is the central hub for secretory cargo protein sorting and trafficking to the plasma membrane. The TGN membrane is highly dynamic and composed of distinct subdomains which differ in their protein and lipid composition. Ultrastructural and real-time imaging studies have provided evidence for the existence of fission domains or hotspots [1–4]. At these sites, vesicular and tubular carriers destined for the plasma membrane or the endolysosomal system emerge from the TGN (Fig. 1A). These fission domains are enriched in cargo and decorated with proteins of the vesicle fission machinery but do not contain any Golgi resident proteins [3,5]. A prerequisite for the existence of these fission hotspots is the segregation of cargo from Golgi resident proteins as well as a proper separation of apical and basolateral cargo to maintain cell polarity. Indeed, studies in polarized cells have revealed that proteins and lipids destined for apical and basolateral plasma membrane leave the TGN at different subdomains [6–9] (Fig. 1A). At these subdomains, a tight coordination of sorting and segregation of cargo protein as well as the specific recruitment of the vesicle fission machinery is required to ensure proper loading of cargo protein into different membrane carriers. In line with this, Golgi-associated proteins such as small GTPases, kinases and phosphatases, lipid transfer proteins, phospholipases as well as membrane-bending and molecular motor proteins show divergent localization within the TGN and contribute to the segregation of fission domains and the maintenance of the dynamic TGN structure [1]. The lipids of the TGN membrane actively participate in this process through the recruitment of cargo proteins and the Golgi-associated vesicle fission machinery thereby coordinating sorting and trafficking, respectively. In addition to this signaling and organizational role, lipids also directly facilitate local membrane curving occurring during vesicle fission and tubule formation. Consequently, the pathways regulating the synthesis and consumption of lipids impact TGN morphology and functionality. Among the major lipids present in the TGN bilayer are phosphatidylinositol (PI) and its phosphorylated derivates [the phosphoinositides (PIP)], glycerolipids, sterols, and sphingolipids (SL; Fig. 1B). In this review, we will discuss how these lipids and their effectors synchronize sorting and trafficking of secretory cargo through a complex network of positive and negative feedback mechanisms.

## Synthesis and localization of glycerolipids, PI, and its phosphorylated derivates, sterols, and sphingolipids

The predominant lipid class in mammalian cells are glycerophospholipids (GPLs) that are the main building blocks of cell membranes. All derive from a glycerol backbone whose 1- and −2- hydroxy groups are esterified with one fatty acid and whose 3-hydroxy function is esterified with phosphate or a phosphoryl alcohol moiety constituting the lipid polar head group.

**Fig. 1.**
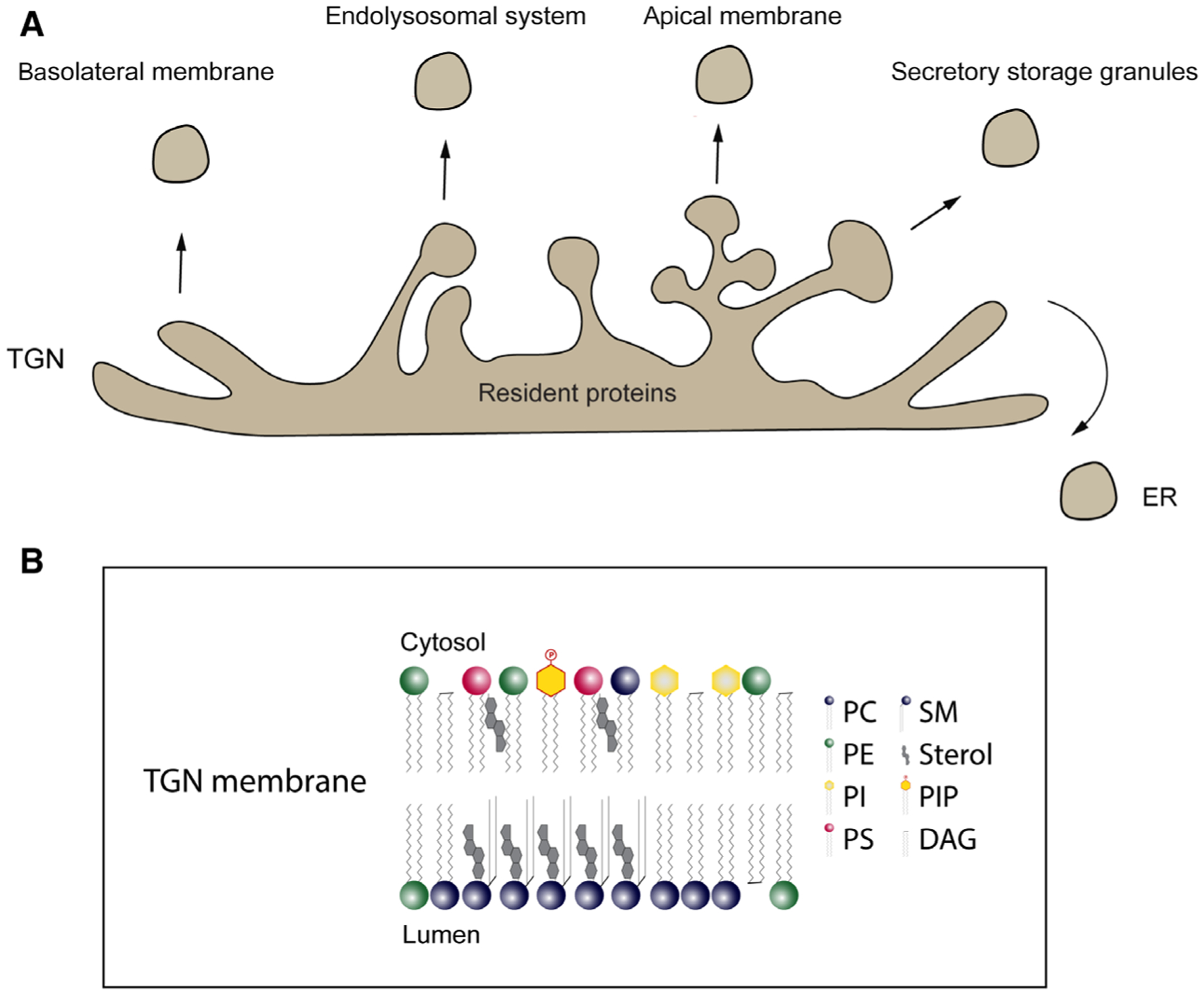
Vesicular biogenesis and transport at the TGN. (A) Graphical overview of the vesicular and tubular network of the TGN including cargo sorting and export domains and examples of final destinations of cargoes (basolateral membrane, endolysosomal system, apical membrane, secretory storage granules, ER). (B) Simplified depiction of the lipid content and topological distribution of lipids in the membrane.

The most abundant subspecies are phosphatidylcholine (PC), phosphatidylethanolamine, PI, phosphatidylserine, and phosphatidic acid (PA). The hydrophobic portion of these species is a diacylglycerol (DAG) with saturated or cis-unsaturated fatty acyl chains with different lengths. PA esterified with an inositol results in PI. The synthesis of GPL subspecies occurs in the endoplasmic reticulum (ER) from PA, which is activated via unique pathways. The combinatorial phosphorylation of the 3-, 4-, and or 5 hydroxy groups of the inositol ring of PI gives seven singular PI phosphates (Fig. 1B). Phosphorylated PI molecules, also termed PIPs, mark cellular membranes and recruit cytosolic proteins [10]. PIPs have a particular important role in controlling membrane traffic and often act in concert with small GTPases from the ADP-ribosylation factor (Arf) and Ras-related in brain (Rab) family and this interplay is essential for Golgi function [11,12].

Ceramide is synthesized *de novo* in the ER and is composed of sphingosine and a fatty acid. The synthesis begins with the condensation of palmitate and serine to form 3-keto-dihydrosphingosine by serine palmitoyl transferase. 3-keto-dihydrosingosine is then reduced to dihydroxy sphingosine which is subsequently acetylated to produce dihydroceramide. The final reaction to produce ceramide is catalyzed by dihydroceramide desaturase. Ceramide is then transferred to the TGN by a nonvesicular process mediated by the ceramide transfer protein (CERT). In the Golgi, ceramides are converted to sphingomyelin (SM), glucosylceramide (GlcCer) and then to more complex SL such as GM3, or to ceramide-1-phosphate. GlcCer, the precursor of most glycosphingolipids (GSLs), is transported by vesicular and nonvesicular routes to generate different classes of GSLs [13]. Within this framework, it is essential to mention that the lipid transfer protein four-phosphate adaptor protein 2 (FAPP2) transfers cis-Golgi–localized cytosolic GlcCer via its glycolipid transfer protein domain to the luminal face of the TGN [14,15]. Importantly, inhibition of ceramide synthesis significantly affects secretory cargo trafficking underscoring the essential role of this lipid in Golgi secretory function [16].

**Fig. 2.**
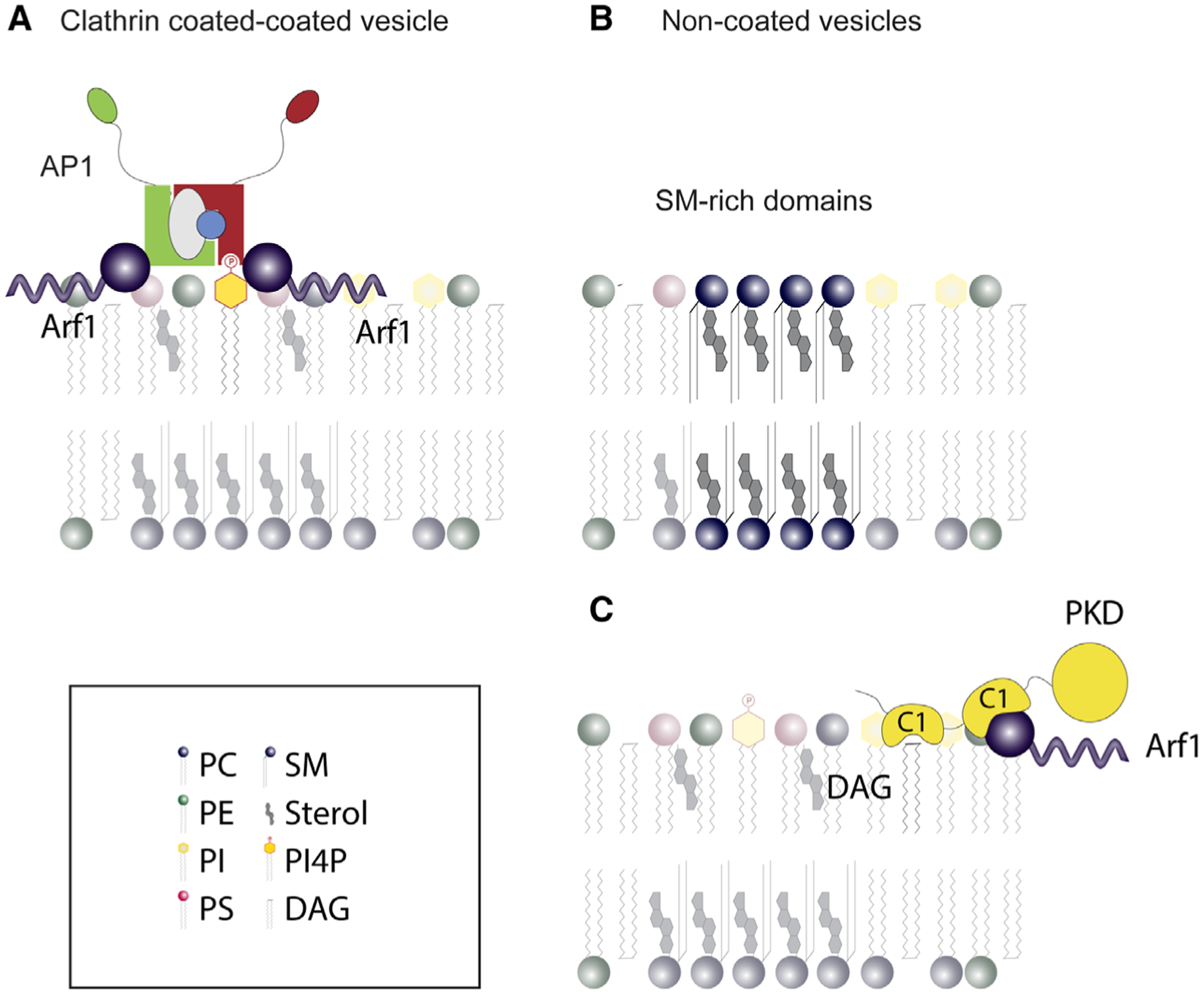
Examples for protein–lipid and lipid–lipid interactions required for the formation of a vesicle at the TGN. (A) Clathrin-coated vesicles: AP1 is recruited by PI4P and Arf1-GTP on the TGN surface to initiate vesicle budding. (B) Noncoated vesicles: Sterol and SL domains constitute sorting domains for vesicular formation. (C) PKD integrates a cysteine-rich zinc finger domain to interact with DAG. Arf1-GTP recognizes a second C1 domain in PKD.

**Fig. 3.**
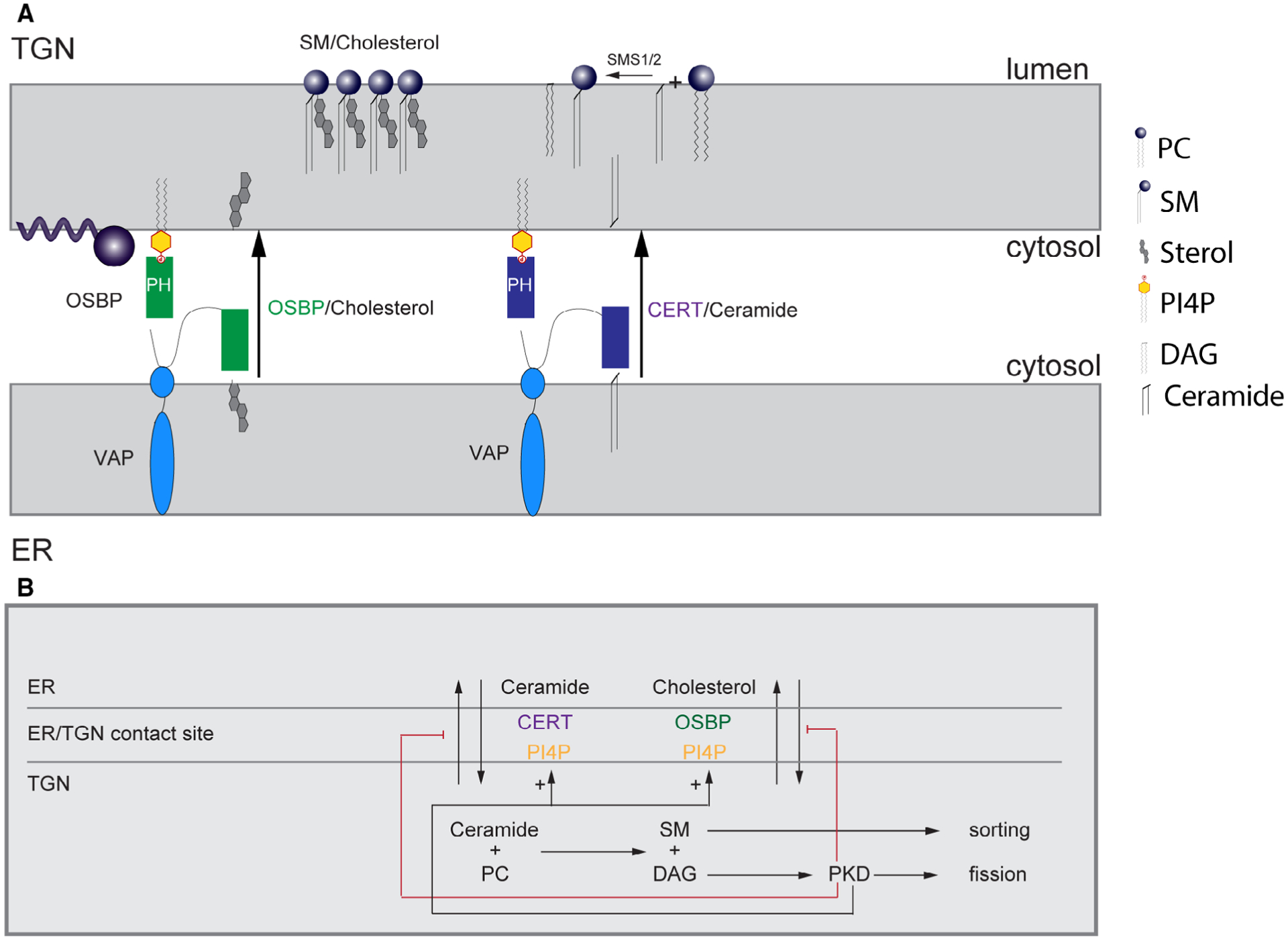
Regulation of lipid transfer at ER/TGN contact sites through positive and negative feedbacks. (A) Oxysterol-binding protein interacts via its PH domain with PI4P and Arf1-GTP and through its FFAT motif with ER-resident VAP receptors. As such, OSBP bridges the ER and TGN membranes and facilitates the recruitment of CERT by PI4P and VAP. CERT delivers ceramide to the TGN. (B) Overview of lipid-dependent coupling of cargo sorting with vesicle biogenesis and fission and its regulation through positive and negative feedbacks at ER-TGN contact sites.

Synthesis of SL such as SM occurs at the luminal leafiet of the TGN membranes by SM synthase 1 (SMS1) and SMS2 converting PC and ceramide to DAG and SM (Fig. 3A) [17,18]. Sterols such as cholesterol are synthesized in the ER via an elaborate multistep process in which the 3-hydroxy-mehtylglutary1-CoA reductase mediates the rate-limiting step catalyzing the conversion of HMG-CoA to mevalonic acid. Sterols are based on a rigid four ring structure that is hydrophobic and structurally distinct from GPLs and SL. Cholesterol contains a hydroxyl group, a steroid, and a hydrocarbon chain which allow the interaction with phospholipids. This interaction reduces the fiexibility of the acyl chains of phospholipids [19,20]. Sterols preferentially associate with saturated PC and SM to generate lateral cohesive contacts with saturated acyl chains to form condensed complexes (Fig. 2B). SL form hydrogen bonds with each other and with the sterol [21]. These interactions allow SL to cluster with sterol into liquid-ordered membrane domains [22]. These domains are reported to function as platforms for protein transport and signaling and are suggested to play a specific role in events leading to transport carrier biogenesis [23].

## Protein and lipid-based cargo sorting at the TGN

The TGN plays a major role in sorting and polarized trafficking of biosynthetic cargo such as transmembrane cargo or proteins attached to membranes, for example by glycosylphosphatidylinositol anchors [1,6]. Several transmembrane proteins have been shown to be sorted by adaptor proteins such as AP1 that recognize sorting signals in their cytosolic domains to recruit clathrin and accessory proteins to the TGN membrane (Fig. 2A) [24]. These motifs have been identified for proteins directed to the endosomal system and for some basolateral-directed cargoes [25–27]. Sorting and trafficking of soluble lysosomal hydrolases occurs by the cation-independent mannose phosphate receptor that recognizes soluble lysosomal pro-enzymes modified with mannose 6-phosphate to elicit their packaging into clathrin-coated vesicles that mediate TGN-to-endosome trafficking [28]. Sorting and generation of vesicles that mediate secretion and the mechanisms that coordinate cargo selection, carrier budding, and fission are poorly understood [29,30]. Simons and colleagues have shown that there is a lipid-based system for cargo sorting and vesicular biogenesis at the TGN independent of coat and adaptor protein-mediated sorting processes [31]. These sorting domains or lipid rafts are small dynamic assemblies within the TGN membrane based on the association between SL and sterols and responsible for the targeting of a subset of cargo molecules that have a particular affinity for liquid-ordered domains [1,22,32,33]. The hypothesis is that these domains cluster membrane cargo proteins to segregate them for transport to apical or basolateral cell surfaces in polarized epithelial cells [33,34]. Transport vesicles are then generated by line tensions at the phase boundaries of the lipid rafts (domain induced budding) [35,36]. These vesicles are rich in SL and cholesterol indicating that lipid sorting occurs at the TGN membrane [34]. The precise mechanisms how these protein lipid assemblies form and regulate TGN sorting of various cargo proteins, however, are still a matter of debate.

## The Sphingomyelin-SPCA1-Cab45 network

Recent studies have uncovered a Ca^2+^-dependent sorting process that is mediated by the TGN-resident Ca^2+^ ATPase secretory pathway ATPase1 (SPCA1). SPCA1 pumps Ca^2+^ into the lumen of the TGN in an ATP-dependent manner [37–39]. The Golgi complex can be considered as a Ca^2+^ store as it can accumulate, store, and release Ca^2+^ and actively participates in Ca^2+^ signaling [40,41]. However, the concentration of Ca^2+^ in the Golgi apparatus is heterogeneous, and it was suggested that there is a Ca^2+^ gradient across the secretory pathway from the ER to the TGN [39]. At steady state, the ER has the highest Ca^2+^ concentration (400 μm), whereas the cis-Golgi contains 250 μm and the TGN around 100 μm of Ca^2+^. TGN Ca^2+^ uptake relies solely on SPCA1 [42]. Using purified proteins, it was shown that the SPCA1 phosphorylation domain, crucial for pump activation, interacts with F-actin in a cofilin-dependent manner [43]. Furthermore, mutation of four amino acids in SPCA1 representing the cofilin binding site affected Ca^2+^ import into the TGN and secretory cargo sorting [43]. What is the role of Ca^2+^ in sorting of secretory proteins at the TGN? The luminal Golgi protein Cab45 that binds incoming Ca^2+^ via its EF-hand domains is a central sorting regulator downstream of SPCA1. Upon TGN Ca^2+^ infiux it oligomerizes and concentrates bound proteins (clients) from the bulk milieu prior to its export from the TGN [44,45]. The identification of TGN derived SM-rich vesicles uncovered an exciting link between synthesis of SL and Cab45-dependent sorting [46,47]. Purification and analysis of these vesicles by a proteomics approach identified Cab45 as one of the most abundant native cargoes of this pathway. Further investigation showed that Cab45 and its client proteins are sorted into SM-rich vesicles.

Interestingly, SPCA1, SMS1, and SL populate the same regions of the TGN. SPCA1 Ca^2+^ pumping activity is promoted by maintenance of a physiologic level of SM in the TGN membranes. In line with this finding, a systematic survey of SPCA1 activity in reconstituted proteoliposomes of differing lipid composition showed that SPCA1 activity is highest in vesicles containing SM [48]. Local hotspots of SPCA1 activity, linked to synthesis and local enrichment of SM within the TGN membrane, will define TGN sorting domains and cargo exit sites. At present, the mechanism by which the SM-rich membrane facilitates SPCA1-mediated calcium pumping is unknown and it may be that SM acts as an agonist by binding to a site(s) on SPCA1 to activate Ca^2+^ pumping, as shown for the paddle domain of plasma membrane voltage-gated K^+^ channels which is critical to voltage sensing [49–52]. These results propose that chemical and physical coupling of SM synthesis, DAG production, Ca^2+^ infiux, and capture of secretory cargo by Cab45 promotes engulfment of oligomeric Cab45-client complexes by the TGN membrane, leading to vesicle budding. SPCA1 activity and cargo sorting are coupled to F-actin dynamics via cofilin on the cytosolic face of the TGN [43,53]. Although it is clear that Ca^2+^ is feeding this process, its source is still unknown. An intriguing possibility would be the idea of membrane contact sites acting as a platform for local Ca^2+^ signaling between the ER and the TGN. Indeed, it is well established that membrane contact sites facilitate Ca^2+^ transfer from the ER to mitochondria. Here, cytosolic glucose-regulated protein 75 connects voltage-dependent anion channel on the outer mitochondrial membrane to the inositol trisphosphate receptor (IP3R) in the ER facilitating Ca^2+^ infiux into the mitochondria [54]. Contact sites between ER- and trans-Golgi cisternae have been visualized by 3D reconstruction of fast freezing and HVEM tomography [55]. Also, these structures seem to be very dynamic and sensitive to Ca^2+^ depletion [56], further supporting the idea that ER-TGN contact sites form close to SPCA1 to promote local TGN Ca^2+^ infiux. What is the role of the dynamic SPCA1-cofilin-F-actin complex? Interestingly protein kinase RNA-like ER kinase, independent of its function in unfolded protein response, regulates ER Ca^2+^ infiux by coordination of ER-PM contact sites through cortical F-actin remodeling by Filamin A [57]. In this respect, it would be an interesting hypothesis that ER-TGN contact sites could mediate binding of cofilin to SPCA1. SPCA1 connected cofilin could depolymerize F-actin in close proximity to allow tethering of ER and TGN for the generation of a Ca^2+^ signaling microdomain. Notably, many proteins involved in Ca^2+^ signaling have been found to be present in SL and cholesterol-rich domains or lipid rafts [58], suggesting that these domains have a significant role in modulating Ca^2+^ signaling. Given the existing link between SL and Cab45-dependent sorting, one could speculate that the formation of lipid rafts in the TGN membrane is required for SPCA1 activation. In support of this idea is the finding that SPCA1 accumulates in cholesterol-rich membrane domains, and its activity seems to be dependent on cholesterol as well [59]. The investigation of these hypotheses, however, is challenging as both ER-TGN contact sites and lipid rafts are small and dynamic and difficult to visualize by fluorescence microscopy in living cells [60]. Also, matching their association with either Ca^2+^ influx or cargo trafficking is very demanding. To overcome these caveats high-resolution live cell microscopy techniques in combination with newly developed fluorescent probes have to be applied. For instance, the laboratory of Akihiko Nakano has established a three color, 4D high speed, and high-resolution microscopy technique that will allow visualizing these dynamic processes in future [61].

## The central role of phosphatidylinositol (4) phosphate in coupling sorting and trafficking of secretory cargo

The main phosphoinositide in the Golgi complex is PI4P, which is generated from PI by the activity of two PI4-kinases (PI4Ks) localized to the Golgi complex in mammalian cells (type III PI4Kβ and type II PI4Kα; [62,63]). While PI4KIIIβ is recruited through binding to the small GTPase Arf1 and the myristoylated Ca^2+^ sensor NCS-1 to TGN membranes [64–67], PI4KIIα requires cholesterol-dependent palmitoylation for interaction with and activation at TGN membranes [68]. The precise distribution of PI4KIIIβ and PI4KIIα within the Golgi complex and thus their specific contribution to the TGN PI4P pool is still not fully understood. From biochemical and localization data, the existence of distinct Golgi pools of PI4P, which might be involved in individual trafficking pathways at different levels, has been suggested [63]. Irrespective of their exact localization, there is plenty of evidence that both kinases, PI4KIIIβ and PI4KIIα, significantly contribute to Golgi PI4P levels and are required for efficient secretory cargo transport [62,67,69–71]. Microscopic detection of PI4P using either protein domains or antibodies that can be labeled with fiuorescent probes revealed that PI4P is specifically enriched at the TGN [63,72]. At this site, it serves as a platform for the association of cytosolic adaptor proteins and components (e.g., arfaptins, GOLPH3, and AP1/clathrin) that aid in deforming the membrane to promote carrier budding and fission [62,73–76]. Moreover, PI4P drives nonvesicular ceramide and sterol transfer from the ER to the TGN thereby controlling SL synthesis [14,18,73,77,78]. PI4P and SL metabolism at the TGN are intimately linked and coordinated through several positive and negative feedback loops. The key mediators in this process are the PI4P effector proteins and lipid transfer proteins CERT [18] and oxysterol-binding protein (OSBP) [79]. Binding of CERT and OSBP to the TGN is mediated through their PH domain which targets PI4P at ER-TGN contact sites where both organelles come into close vicinity (Fig. 3B) [64,72,80]. Likewise, a FFAT motif in both proteins is responsible for binding to the integral membrane protein VAMP-associated protein A/B (VAP-A/B) in the ER [81–83]. While CERT transfers ceramide from the ER to the TGN for the production of DAG and SM through the activity of SMS1 [17,18], OSBP tethers the ER and TGN membranes at these contact sites for nonvesicular exchange of cholesterol and PI4P and facilitates CERT-mediated ceramide transfer [64,84,85]. Thus, by removing PI4P, OSBP negatively feeds back on its TGN localization and consequently ER-TGN membrane tethering and lipid transfer. Disrupting the binding of CERT and OSBP to VAP-A/B, depleting VAP-A/B isoforms or CERT and OSBP substantially impaired the processing and secretion of secretory cargo demonstrating the importance of lipid transfer at ER-Golgi contact sites for secretory cargo trafficking [82,86]. In the ER, OSBP-transported PI4P is immediately consumed by the phosphatidylinositol phosphatase Sac1, an integral ER membrane protein [70,87]. This process is proposed to provide the energy for OSBP-mediated cholesterol transport from the ER to the TGN [64]. A recent study by the group of Antonella de Matteis provides further evidence that the PI4P-and Arf1 binding protein FAPP1 positions Sac1 at ER-TGN contact sites thereby locally restricting PI4P consumption and consequently secretion. In this respect, FAPP1 acts also as a PI4P sensor [88]. At ER-TGN contact sites Sac1 forms a complex with OSBP and VAP-A/B [86]. To maintain the PI4P gradient on Golgi membranes with PI4P being high at the TGN and low at cis/medial Golgi membranes Sac1 also traffics between the ER and the early/cis-Golgi membranes through COPI-and COPII-dependent vesicular transport; however, the authors reported Sac1 to be absent from TGN membranes [89–91]. These at first glimpse conflicting data could be explained by the highly dynamic nature of ER-TGN contact sites which are supposed to continuously form and dissolve making their visualization challenging [92]. However, through high-resolution electron microscopy combined with a newly developed FLIM-FRET imaging approach, de Matteis and her group revealed the presence of Sac1 at these sites in their native state [93]. Depletion of Sac1 results on one hand in accumulation of PI4P in ER membranes [94], and on the other hand to the loss of the graded distribution of PI4P between Golgi compartments [90] confirming both modes of action for Sac1. The net effect of Sac1 depletion is an overall grossly disturbed Golgi morphology and a missorting of Golgi-resident glycosylation enzymes [90] demonstrating the importance of a tightly organized distribution of PI4P at TGN membranes for proper sorting and vesicle budding.

## The DAG-PKD network and its functional relevance for transport carrier fission

The lipid metabolite DAG has a central role in maintaining Golgi structure and vesicle budding and fission [95]. DAG levels are determined by different metabolic pathways which regulate its production or consumption. The main production pathway for DAG at the Golgi complex is catalyzed by SMS1 which transfers phosphocholine from PC to ceramide to generate DAG and SM (Fig. 3) [96]. Additionally, DAG is also generated by PA phosphatase (PAP), which dephosphorylates PA [97,98]. Further studies provided evidence that the family of PI-specific phospholipase C (PI-PLC) enzymes, which generate DAG and IP3 through the hydrolysis of PI(4,5)P_2,_ are present at Golgi membranes. Within the PI-PLC family, three members, PLCβ3, PLCγ1, and PLCε, have been reported to localize to the TGN and to participate in membrane trafficking [99–102]. PLCβ3 is activated by binding of Gβ/γ proteins to its PH domain [103] whereas PLCε activation can also occur through Rho GTPases [104]. Notably, the hydrolysis of PI4P has been demonstrated for all mammalian PI-PLC isoforms *in vitro* [105]. Indeed, a recent report provides evidence that PI4P is the major source of biologically relevant DAG production [106]. However, so far the regulation of these pathways and their contribution to the production of DAG at the Golgi complex under physiological conditions is not fully understood. An attractive hypothesis is that the arrival of cargo proteins activates DAG synthesis pathways [107]. Indeed, cargo arrival at the Golgi initiates a signaling circuit necessary for the Golgi-to-plasma membrane transport [108] and activates PLCγ1 to locally increase DAG levels [101]. Additionally, extracellular signaling from G protein-coupled receptors (GPCRs) has been shown to activate PLCε at Golgi membranes. As a result, PI4P is hydrolyzed to generate DAG and inositolbisphosphate [100,102]. However, how extracellular signaling and cargo arrival are synchronized to induce DAG synthesis and thus the assembly of the fission machinery in a timely manner is still a mystery.

Diacylglycerol consumption is predominantly mediated by the cytidine diphosphate (CDP)-choline pathway for PC biosynthesis and this contributes significantly to the budding and fission of vesicles [109,110]. In this respect, it is important to mention that the lipid transfer protein Nir2 exchanges PC for PI at the ER-TGN interface thereby critically participating in PI4P synthesis and DAG levels at the TGN [82,109]. Aside from this, the consumption of DAG is also regulated by DAG kinase, which phosphorylates DAG to generate PA, or by SMS1, which converts DAG and SM back to PC and ceramide. The contribution of these pathways to the secretory function of the Golgi complex is, however, not sufficiently understood. At the TGN, DAG fulfills several functions. First, DAG can facilitate the negative curvature needed for membrane fission [111]. Second, it serves as a signaling scaffold to assemble the fission machinery. Here, DAG is essential for recruitment of protein kinase D (PKD) to the TGN but also for the activation of the kinase at this compartment [16]. The three PKD isoforms PKD1, 2, and 3 comprise a family of serine/threonine kinases those activity is required for the fission of exocytic carriers at the TGN named CARTS (carriers of the TGN to the cell surface) [112–117]. Notably, Arf1 co-operates with DAG in TGN localization of PKD [118]. Inhibition of PKD activity (by siRNA-mediated depletion, pharmacological inhibition or expression of a dominant-negative PKD mutant) results in the formation of tubular structures which contain cargo protein but are not able to detach from TGN membranes [102,113].

At the TGN, PKD phosphorylates and activates PI4KIIIβ [119] thereby contributing to PI4P production and consequently, recruitment of CERT and OSBP [18,120]. The phosphorylation of PI4KIIIβ also induces its complex formation with 14-3-3γ proteins [121], PAK1, Arf1, and BARS [77]. BARS itself binds to and activates a trans-Golgi lysophosphatidic acid (LPA) acyltransferase type δ that converts LPA into PA [122]. While it is clear that this reaction is essential for the fission of basolateral carriers the fate of PA is less well understood. PA could be dephosphorylated to generate DAG, which would amplify the recruitment and activation of PKD. Indeed, inhibition of PAP activity by propranolol impairs PKD localization [16] and membrane bud formation [98]. In further support of this idea is the finding that the catalytic activity of PLA_2_, which cleaves phospholipids to generate free fatty acids and LPA, is required for PKD localization to the TGN [123]. It is, however, also conceivable that the DAG generated is used for the production of PC through the CDP-choline pathway to terminate the assembly of the fission-inducing complex. Although the molecular mechanisms are still unclear, there is sufficient evidence that continual phospholipid remodeling by various enzymes is critical to regulation of the availability of curvature-altering lipids such as LPA, PA, and DAG. The net effect of PKD recruitment to and activation at TGN membranes is thus to promote these local changes in the lipid composition which facilitate the negative membrane curvature but also lead to the assembly of the fission machinery.

Strikingly, PKD also directly phosphorylates CERT and OSBP thereby mediating their release from PI4P and Golgi membranes allowing for further rounds of lipid transfer [124,125]. This negative feedback loop is thus supposed to maintain DAG, SM, and cholesterol levels at the TGN and provides a direct link between SL synthesis, exocytic cargo sorting, and carrier formation. Indeed, the regulated production and especially the organization of SM is essential for Golgi cisternae morphology, the biogenesis of transport carriers at the Golgi membranes, and proper cargo glycosylation [23,126,127]. A recent publication by Capasso and co-workers demonstrates how these feedbacks act to buffer acute fluctuations in SL production thereby maintaining SM levels for proper carrier formation. Under conditions of low SL flow, CERT transports ceramide efficiently from the ER to the TGN to produce SM and DAG. High-SL flow through increased ceramide transfer enhances SM production. This triggers PKD-mediated OSBP phosphorylation (potentially through in parallel enhanced DAG levels) and in turn PI4P turnover at the ER through Sac1 [128]. As a net result of PI4P consumption, CERT-mediated ceramide transfer and SM production are dampened.

The spatiotemporal connection of secretory cargo sorting and vesicle fission is further supported by the finding that local SM synthesis promotes Ca^2+^ influx into the TGN lumen. This process couples SM metabolism with Cab45-dependent cargo sorting [46]. Notably, several Cab45 clients are associated with CARTS whose formation is dependent on the DAG/PKD network [116,129]. PKD in turn is also involved in SM synthesis through the regulation of CERT localization to the TGN [124]. Moreover, the formation of CARTS is dependent on VAP-A/B controlled lipid transfer through CERT and OSBP at ER-Golgi contact sites [86]. This complex system of negative and positive feedback loops ensures the position and timely assembly of SM and cholesterol-rich membrane domains with the fission machinery (Fig. 3B). In this respect, the ER-Golgi contact sites might represent ‘hotspots’ of secretory cargo sorting and membrane fission.

## Coupling the cell environment to lipid metabolism and Golgi secretory function

It is well known that growth factors stimulate the delivery of proteins and lipids to the plasma membrane but how signals generated at the plasma membrane initiate sorting and fission of cargo at the TGN is still not fully understood. The first hint for integration of growth factor signaling and Golgi secretory function was provided by the observation that cycling of Sac1 between the ER and the Golgi complex is growth factor dependent. This allows the cell to properly adapt its secretory function to external cues [130]. Specifically, Sac1 oligomerizes and accumulates in the Golgi in quiescent cells resulting in a sharp decrease of PI4P and constitutive secretion. After growth factor stimulation, p38 MAPK activity is required for the dissociation of Sac1 complexes, which triggers retrograde traffic and redistribution of Sac1 to the ER. This in turn shifts the balance toward PI4K-mediated production of PI4P thus accelerating constitutive secretion [130]. This mechanism of nutrient sensing and transmitting raises the challenging question whether localization of Sac1 to ER-TGN contact sites or, more importantly, their dynamic formation and tethering through OSBP is sensitive toward growth factor stimulation as well. Indeed, ER-mitochondria contact sites respond to nutrients such as glucose thereby controlling mitochondria function [131]. We recently uncovered a signaling pathway that connects GPCR signaling at the plasma membrane with PLCε-mediated DAG production, PKD activation and vesicle fission at the TGN [102]. Notably, the localization of the ER-Golgi tethering protein OSBP is regulated by PKD-mediated phosphorylation [124,125] underpinning a potential regulation of ER-Golgi contact sites through extracellular cues. This also is in favor of the idea of ER-Golgi contacts being not only physical conduits for lipid exchange between organelles but rather serving as regulatory interfaces to integrate lipid synthesis pathways with secretory cargo sorting and trafficking. High-resolution live cell imaging of the ER contact sites in their native state will be needed to resolve these important questions.

Cells sense the mechanical properties of the extracellular matrix (ECM) through their integrin receptors. To properly adapt to the extracellular conditions such as stiffness and ECM topography cells respond by changing their actomyosin cytoskeleton contractility and inducing signaling pathways. While it is intuitively clear that mechanical cues and the secretory pathway must be linked to ensure proper cell function, it is still not fully understood how cells transduce mechanical forces to coordinate Golgi secretory function. Some recent studies shed more light on this question: Singh and co-workers showed that Golgi complex integrity and Arf1 activity are dependent on integrin-mediated adhesion [132]. Specifically, the authors report on an integrin-activated Arf1–dynein–microtubule pathway that controls adhesion-dependent Golgi organization. Mechanistically, the ArfGEF BIG1/2 was proposed to be required for Arf1 activation; however, the molecular mechanism of how integrins signal to BIG1/2 at TGN membranes remains unclear. Strikingly, proper cell surface protein glycosylation relied on adhesion-dependent Golgi organization in an Arf1-dependent manner thereby connecting mechanical cues with Golgi function. Considering the important role of Arf1 in secretory cargo trafficking through the recruitment of effector proteins such as golgins, PKD, and FAPP2 [67,73,118,133], loss of adhesion-mediated regulation of the Golgi and, particularly, the TGN is expected to affect Golgi-dependent secretory cargo sorting and trafficking. Romani and co-workers went one-step further and established the Golgi complex itself as a mechanosensor organelle [134]. They show that the Golgi complex responds to intracellular and extracellular mechanical forces such as actomyosin contractility and matrix stiffness, respectively, through alterations in DAG levels. The molecular pathway connecting these mechanical forces with lipid metabolism is composed of the PAP lipin-1 that converts PA into DAG [135]. Inhibition of lipin-1 by placing cells in a soft matrix sharply decreased DAG levels proving that lipin-1 activity is sensitive toward actomyosin contractility. As a result, Arf1 levels at Golgi membranes dropped [134]. This suggest that local changes in PA and DAG could impinge on Arf1 function. Indeed, lipin-1 has been described previously to be upstream of Arf1 activation potentially through regulating the localization of the ArfGEF GBF1 to Golgi membranes [136]. *In vitro*, PA synergistically potentiated the phosphoinositide-dependent activation of ArfGAP proteins such as the Arf1GAP AGAP1 [137]. Thus, it is likely that the PA regulation of an ArfGAP is a common mechanism for the inactivation of Arf small GTPases. Irrespective of the mechanism of how ARF1 activity is controlled by PA and DAG, the loss in ARF1 activity negatively affected the trafficking of transcription factors sterol regulatory element binding protein family 1 and 2 (SREBP1 and 2) between the ER and the Golgi. SREBP1 and 2 are activated by protease-mediated cleavage in the Golgi to promote the lipogenesis of cholesterol, fatty acids, PC, and triglycerides [138]. As a consequence, matrix stiffness and actomyosin contractility directly control cellular lipid metabolism through the Golgi complex [139].

Although stiffness-dependent effects of lipin-1 on the secretory pathway at the level of the TGN were not investigated, a role for this pathway in DAG dependent membrane budding and secretion becomes apparent. Along these lines, Wakana and co-workers found just recently that SREBP-cleavage-activating protein (SCAP), a cholesterol sensing protein in the ER that escorts SREBPs for their export from the ER to the Golgi [138], is required for the biogenesis of CARTS at ER-Golgi contact sites [140]. While low cholesterol levels in the ER trigger the export of SCAP/SREBPs to the Golgi complex, high-cholesterol levels retain SCAP in the ER through interaction with the integral ER membrane protein Insig (Insulin-induced gene protein) [141,142]. ER-retained SCAP subsequently builds a complex with Sac1 and the VAP/OSBP complex at ER-Golgi contact sites to promote lipid transfer and the formation of SM and cholesterol-rich membrane domains [140].

Extracellular matrix stiffness was also found to destabilize microtubules, releasing GEF-H1, which was required for the invasion of breast cancer cells through 3D matrices [143]. We have recently shown that GEF-H1 is upstream of PKD-controlled vesicle fission at the TGN [102]. Given that PKD localization and activity is dependent on the amount of DAG in TGN membranes, it is intriguing to speculate that the cytoskeleton, either through Rho-mediated activation of Golgi-localized PKD, or lipin-1-mediated DAG production senses the ECM density to coordinate Golgi secretory function with the increased demand for factors such as proteases or cytokines required for ECM remodeling during 3D migration.

## Conclusions and perspectives

In past years the list of lipids, lipid-modifying enzymes and lipid effector proteins involved in secretory cargo sorting and trafficking at the TGN has been quickly growing (Table 1). The fact that these lipids and proteins are engaged in a complex system of negative and positive feedback loops likely ensures the synchronization of secretory cargo sorting with initiation of vesicle fission. The components of the sorting and fission machinery assemble at ER-Golgi contact sites which may present specialized zones for secretory cargo trafficking to the plasma membrane. Golgi PI4P has a central role in coupling secretory cargo sorting and trafficking as it, through its effector proteins, orchestrates the spatial and temporal assembly of the key lipids SM and DAG required for activation of the sorting and fission machinery.

There are, however, still many open questions pertaining to the investigation of these key components and membrane domains that remain to be investigated. Do different local pools of PI4P, generated either by PI4KIIIβ or PI4KIIα, exist in TGN membranes? If so, does each pool have different effectors and how is this regulated? Does cholesterol contribute to Cab45-based protein sorting? How is signaling to and from the Golgi complex coordinated to synchronize extracellular demands with lipid-dependent intracellular cargo sorting and trafficking? Are ER-Golgi contact sites representing ‘hotspots’ of secretory cargo sorting and vesicle fission and do extracellular cues control their dynamics through the cytoskeleton? The identification of proteins which can be used as markers for fission hotspots at the TGN would be a prerequisite to address these questions. A potential candidate could be Rab6, which is required for the formation of various transport intermediates at the TGN and decorates post-Golgi carriers [2,144]. On the technical side, the visualization of the spatiotemporal distribution of various lipid species by high-resolution imaging in living cells using novel specific probes [145,146] in combination with multicolor microscopic analyses of cargo sorting and trafficking [61] will help to resolve some of these important questions. Additionally, the field of mathematical modeling is increasingly exploited to address different aspects of lipid function at the TGN. These models will aid in providing predictions for reactions difficult to measure experimentally, for example lipid metabolic flow and transfer rates [111,126,147,148]. Here, the development of new methods that combine full-lipidome quantification with simultaneous monitoring of the time-dependent turnover of lipid species such as shotgun ultra high-resolution mass spectrometry [149] will be of special importance.

Finally, although research has extended our knowledge on how lipids control secretory cargo sorting and trafficking at the molecular level, the significance of these findings *in vivo* is largely unclear. Generation of knock-out mice to study the relevance of lipid metabolism on Golgi secretory function might be helpful to address this question; however, as most of these enzymes have multiple intracellular locations and functions beyond Golgi lipid homeostasis, the interpretation of phenotypes with respect to Golgi function will be challenging.

## Figures and Tables

**Table 1.** Golgi proteins and their interacting lipids in secretory cargo sorting and trafficking.

Protein	Interacting lipid	Function	Reference
OSBP	PI4P	Recruitment of OSBP to TGN membranes through PI4P	[64,70,72,120,150]
	Cholesterol	Nonvesicular exchange of PI4P and cholesterol between the ER and the TGN	
CERT	PI4P	Recruitment of CERT to TGN membranes through PI4P	[18,151]
	Ceramide	Nonvesicular transport of ceramide from the ER to the TGN	
FAPP1	PI4P	Recruitment of FAPP1 to TGN membranes through PI4P - activation of Sac1	[73,75,76,88]
FAPP2	PI4P	Recruitment of FAPP2 to TGN membranes through PI4P	[13,14,73]
	GlcCer	Nonvesicular transport of GlcCer from the cis-Golgi to the TGN	
SCAP	Cholesterol	Promotion of conformational change and interaction of SCAP with Insig in the ER membrane	[141,142,152]
PKD	DAG	Recruitment to and activation of PKD at TGN membranes	[16]
SPCA1	SM	Activation of SPCA1	[46]

## Abbreviations

Arf: ADP-ribosylation factor
CDP: cytidine diphosphate
CERT: ceramide transfer protein
DAG: diacylglycerol
ECM: extracellular matrix
ER: endoplasmic reticulum
FAPP2: four-phosphate adaptor protein 2
GlcCer: glucosylceramide
GPCR: G protein-coupled receptor
GPL: glycerophospholipid
IP3R: inositol trisphosphate receptor
LPA: lysophosphatidic acid
OSBP: oxysterol-binding protein
PA: phosphatidic acid
PAP: PA phosphatase
PC: phosphatidylcholine
PI: phosphatidylinositol
PI4K: PI4-kinases
PIPs: phosphoinositides
PI-PLC: phosphatidylinositol-specific phospholipase C
PKD: protein kinase D
Rab: Ras-related in brain
SCAP: SREBP-cleavage-activating protein
SL: sphingolipid
SM: sphingomyelin
SMS1: SM synthase 1
SPCA1: secretory pathway ATPase1
SREBP: sterol regulatory element binding protein family
TGN: trans-Golgi network
VAP-A/B: VAMP-associated protein A/B
